# Supplementary material for: Visuomotor adaptation across the lifespan
Source: PLoS One. 2024 Jul 11;19(7):e0306276. doi: 10.1371/journal.pone.0306276 (PMC11238954; doi:10.1371/journal.pone.0306276)
Supplement: S1 File — (PDF) [file pone.0306276.s001.pdf]

Learning rates

In order to assess the speed at which participants in each age category adept, de-adapt and re-adapt, we fit 3 exponential functions to the learning (or unlearning) curves. For the case of adaptation and re-learning, we use this equation:

$$x = a - \left(a \cdot (1 - l)^t\right)$$

Where x is the predicted set of reach deviations, t is a sequence of trials with the first trial having number 0, and two free parameters: a is an asymptote and l is a learning rate. For washout (or unlearning), we fit this equation:

$$x = s \cdot r^t$$

Which works almost the same way, but here we have a starting level s, and a retention rate r, that is used to predict the reach deviations x over time t.

For the initial phase we baseline the data using the last 9 reach deviations in the aligned phase. For relearning, we see that participants didn’t decay back to zero, so we use the predicted value for the trial after the last washout trial as a reference level. This also means that asymptotes are relative to that level, and not relative to zero.

We used bootstrapping (1k samples of the same number of participants as are in each group, with replacement, from all participants in each group) to obtain 95% confidence intervals that could be used as descriptive statistics for comparisons between groups or conditions (these do not account for multiple comparisons though).

In order for the bootstrapped values in the relearning phase to be based on the correct starting levels, the washout and relearning fits were done on the same random samples. The initial learning rates were bootstrapped separately however.

We evaluated the quality of fit using the mean squared error (MSE) between the predictions from the fit and the actual data. In each fit, we first did a grid search on 11x11=121 combinations of parameter values spread evenly through the parameter space, calculating the MSE for those combinations. We used the 10 parameter combinations with the lowest MSE’s as starting points for a quasi-Newton optimization algorithm with box constraints that further reduced the MSE (the rates were restricted to the range [0,1] and the asymptotes and starting value to the range [0,max(data)]). The set of parameters yielding the lowest MSE was used as the optimal fit. This was repeated for each of the 1k samples of participants, to get the confidence interval for each parameter. We got the central value of each parameter by fitting the function to the data from all participants.

For each fit, we only used the data for the 45 degree targets, and averaged across participants to get a single time course to fit.

The central values and 95% confidence intervals of the mean for each parameter can be found in table 1 below.

phase, measure	Age groups YC	OC	YA	MA	OA
**Initial adaptation**					
Learning rate	0.388 [0.296-0.523]	0.297 [0.223-0.395]	0.160 [0.114-0.217]	0.196 [0.158-0.238]	0.223 [0.184-0.277]
Asymptote	37.00° [33.38°-40.57°]	40.27° [38.66°-41.56°]	38.38° [36.75°-40.20°]	41.45° [39.87°-42.94°]	42.71° [40.39°-45.32°]
**Washout**					
Start (after effect)	27.83° [18.35°-37.91°]	34.15° [30.45°-38.01°]	30.18° [22.36°-37.98°]	32.71° [29.40°-35.92°]	34.91° [27.84°-40.76°]
Retention rate	0.948 [0.843-0.993]	0.877 [0.825-0.915]	0.804 [0.610-0.868]	0.942 [0.925-0.957]	0.895 [0.848-0.937]
**Relearning**					
Base level	12.43° [2.15°-24.29°]	4.78° [1.99°-8.45°]	1.15° [0.02°-3.20°]	13.40° [10.42°-16.47°]	6.64° [3.14°-11.64°]
Learning rate	0.600 [0.303-0.873]	0.654 [0.570-0.736]	0.581 [0.452-0.701]	0.490 [0.394-0.598]	0.566 [0.491-0.633]
Asymptote	25.53° [15.57°-34.84°]	34.70° [31.27°-37.28°]	34.34° [30.61°-37.29°]	27.37° [24.46°-30.45°]	33.51° [28.95°-36.93°]

Table 1. Exponential function fits with parameter confidence intervals. *In columns are the different age groups, from youngest to oldest. In rows we find the parameters. The top two rows present values for exponential fits to reach deviations in the initial adaptation block. The next two rows present values for parameters describing exponential decay during the washout/generalization phase. The last two rows represent parameter values fitting the time course of reach deviations in the relearning phase, including the "base level", i.e. the reach deviation predicted by the fit to the washout phase on the first trial of the retention phase. In each cell, we first see the value obtained from a fit to data from the full group of participants, followed by the 95% confidence interval in square brackets, obtained from a bootstrapping procedure.*

Published papers using this method:

Bansal A, ’t Hart BM, Chauhan U, Eggert T, Straube A, Henriques DYP. Motor adaptation does not differ when a perturbation is introduced abruptly or gradually. Exp Brain Res, 2023, doi: 10.1007/s00221-023-06699-2

Ruttle JE, ’t Hart BM, Henriques DYP. Reduced feedback barely slows down proprioceptive recalibration. J Neurophys, 2022, doi: 10.1152/jn.00082.2022

Ruttle JE, ’t Hart BM, Henriques, DYP. Implicit motor learning within three trials. Sci Rep, 2021. doi: 10.1038/s41598-021-81031-y

Pre-print:

Gastrock RQ, ’t Hart BM, Henriques DYP. Distinct learning, retention, and generalization in de novo learning. preprint, 2023, doi: 10.1101/2023.10.02.560506
