# Supplementary material for: Visuomotor adaptation across the lifespan
Source: PLoS One. 2024 Jul 11;19(7):e0306276. doi: 10.1371/journal.pone.0306276 (PMC11238954; doi:10.1371/journal.pone.0306276)
Supplement: Attachment — Submitted filename: Response to Reviewers.docx [file pone.0306276.s002.docx]

Reviewer #1: In this article the authors investigate visuomotor adaptation capabilities across the lifespan (from young children to older adults) using a shortened, gamified task. They report similarities in learning, unlearning, savings, and generalisation between all age groups. Overall, the manuscript is well presented and reasoned, however, there are some concerns that need addressing before suitable for publication.

Major Comments:

1. A main concern with the manuscript lies in the choice of task used and thus the generalisability of results to the wider literature. It is understood that the shortened and gamified version of the task was chosen to keep the task brief, and participants engaged (particularly younger children), however, the decision to only assess adaptation towards one target may have had significant implications on the results, specifically age-related differences. It is likely that task constraints of only adapting to one target would be much less than adapting to four or eight targets, as described in much of the existing literature. This is particularly pertinent, as impaired cognitive/strategic abilities have been attributed to reduced adaptive performance in younger children and older adults. While the benefits of the adapted task are discussed throughout, the potential caveat to the results needs to be addressed in the discussion or a limitations section, especially as real-world adaptive processes involve complex interactions between multiple sensory inputs and motor outputs.

**REPLY:** **Thank you for all your valuable insights and highlighting this concern. Because we wanted to look at generalization, in addition to many other features of motor learning, it was necessary to have participants adapt to only one target during the learning phase, so that we could compare this learning to other targets during washout. Additionally, like you said, only having one target location during learning allowed us to accelerate learning during that phase and to have a shorter experiment overall. While we agree that having more target locations might tap into impaired cognitive/strategic abilities in younger children or older adults, this was not a focus of the current study. We have added a limitations section, to address this along with some of the other concerns you and other reviewers have raised.**

2. The sample sizes for each group differ significantly in many of the age-groups. This is not acknowledged or discussed in the manuscript and no power calculations are included. The authors should at least provide an explanation or rationale for this. They should also report whether the data is normally distributed and adjust analysis appropriately e.g., consider using median scores rather than means.

**REPLY: Since our study has groups that are still larger than most motor learning studies, we originally omitted the results of the power calculation we ran, which indicated we would need at least 40 participants per group. However, such power calculations are only as good as the estimate of effect size, if there is an effect at all, so we usually don’t rely on them, aiming instead to gather as large a sample as possible. Note that after reviewing the literature, we are not convinced that an effect of age should be found when the rotation is small and the task is more engaging, making it difficult to estimate the parameters needed to conduct the power analysis.**

**Regarding normality, we assume you are asking about it since it does appear that some distributions, represented by the boxplots in Figure 3B, are slightly skewed. However, it has been shown in a Monte Carlo simulation (see Blanco et al., 2017) that frequentist ANOVAs are quite robust under various violations of normality, such as unequal sample sizes, across a wide range of total sample sizes, and various shapes of distributions, even when some group distributions are skewed, while others are not (which appears to be the case when looking at Figure 3B). Of course, this is not an issue for Bayesian statistics, which we also report. Given that we wanted to present this paper as simply as possible, to make it accessible for a more general audience who is interested in aging (and may not be experts in motor learning or advanced statistics), we would like to avoid reporting normality scores (and think it is unnecessary given the findings of Blanco et al., 2017 and our Bayesian analyses), especially since we would need to do so for a large number of analyses.**

3. Some aspects of the ‘Data Analysis’ section are written passively, with ‘then’ and ‘next’ repeated multiple times in each subsection making it difficult to follow exactly why each test was run and for what reason. This should be re-drafted throughout, for example, instead of “Then we wanted to confirm that each group showed aftereffects by performing 5 separate single-sample t-tests, for each of the groups, to see if their reach deviation for the first block of washout was significantly different from 0.”, consider changing to something like “To confirm that each group showed aftereffects, we performed 5 separate one-sample t-tests (one for each group), determining whether reach deviation during the first washout block was significantly different from 0.” Etc.

**REPLY: Thank you for this suggestion. The data analysis section has been edited and now uses a more active voice throughout.**

4. Figure 2 should be edited to include individual data points and/or the authors should consider re-plotting as box plots (similar to Figure 3) to improve interpretation of these results.

**REPLY: Including individual data points is a great idea. To retain readability of the paper for a more general audience, we have kept the Figure 2 plots as they currently are but have added scatter plots of the individual data (2 additional panels), which are now beside the respective graphs for accuracy and variability. We chose this format because the box plots in Figure 3B are showing data for sets of 3 averaged trials, with the baseline mean subtracted, whereas Figure 2A is showing the actual baseline means; we think using these different visualization styles helps to better distinguish between these results.**

5. The authors nicely highlight the differences in variability between young children and the other age groups during baseline trials in the discussion. However, they do not attempt to explain this result with evidence from the wider developmental motor control literature. Some reasoning on this matter should be included.

**REPLY: Thanks for catching this; we agree this should have been supported with other literature. There are 2 possible explanations: 1) younger children are less precise in their movement execution, or 2) younger children engage in more exploration of the movement space to solve the task, but we can't distinguish between the two types of explanations with our data. However, we have now referenced Contreras-Vidal et al. (2005), who speculates that more variable motor control in young children is due to less fine-tuned motor abilities (Page 20, lines 426-427). We have also reminded readers of the correlation we found (lines 427-428) between variability and mouse experience in children, and now use this as a possible explanation for our results.**

6. Following on from the previous point, the authors do not carry out (or have not presented results) on variability for the other phases of the task. It seems strange to only conduct this analysis for baseline trials (unless there is a specific reason – which should then be included in the methods). It appears like younger adults are much more variable than the other age-groups during washout trials as well, which may be an interesting point to investigate further. At the very least, a rationale for only looking at variability during baseline reaches needs to be included.

**REPLY: We think you meant to refer to young children here, who do appear to be more variable in the washout phase. We only calculated variability for the baseline phase because it was the only phase where it made sense to do so. In other phases, the performance measure (baseline mean) changes as participants adapt, de-adapt, and re-adapt. Therefore, the standard deviation is not a reliable measure in the other phases. We have now added this explanation to our data analysis section (Page 11, lines 217-220).**


Minor Comments:

1. Line 49: The authors state that “young adults usually adapt quickly and completely”. However, this is not necessarily true. In fact, there is whole body of literature dedicated to understanding why individuals are unable to fully compensate for visuomotor rotations. Please alter the sentence so it reflects the literature.

**REPLY: Thank you for bringing this up. We say “usually”, based on our experience testing a vast number of young adults over the years. You are correct that there are situations in which this does not occur completely. We have edited the sentence to be “young adults usually adapt quickly.” This statement now falls on line 47.**

2. Ruitenberg et al., 2023. Developmental and age differences in visuomotor adaptation across the lifespan. This relatively new paper aims to answer similar questions but finds contrasting results and should be introduced and discussed in the manuscript.

**REPLY: Thank you for suggesting this new paper, which is a valuable source to bring in since it also compares adaptation performance throughout the entire lifespan. We now refer to it in our introduction and speculate on some of the reasons their findings might be different from ours, particularly for children and older adults, throughout relevant parts of our discussion section.**

3. Line 65-67: This sentence feels out of place at this point in the introduction “Most studies focus on the overall magnitude of adaptation, rather than its speed, so we compared changes across blocks of 3 trials to better capture potential group differences in learning rate.” The authors should consider removing it.

**REPLY: We have moved this sentence to the last paragraph of the introduction (Page 5, lines 100-102) where we introduce our main goals.**

4. The authors tend to underrepresent the wealth of evidence suggesting visuomotor adaptation is impaired in older adults – maintaining the line that evidence is relatively balanced with respect to adaptive capabilities. This tone should be altered, and key references should be added: Hardwick & Celnik, 2014; Panouillères et al., 2015; Weightman et al., 2020 (tDCS paers reporting age-related visuomotor impairments in the respective young and older adults sham stimulation groups, with different rotation magnitudes), & Vandervoorde & Orban de Xivry, 2019, J Physiology.

**REPLY: There is indeed evidence that visuomotor adaptation is impaired in older adults for larger rotations, but this is certainly not the consensus (Noohi et al., 2015, Vachon et al, 2020; Vandevoorde & Orban de Xivry, 2019), especially when rotations are smaller (Heuer & Hegele, 2008). We have altered the tone a bit on line 488. As you are correct, the more common opinion is that older adults show impaired adaptation performance, although it is not so clear cut; Vandevoorde & Orban de Xivry (2019) only found differences in older adults when they specifically probed explicit, or cognitive, components of adaptation and they also had a large sample size, like us. It is also possible some studies that did not show the “expected” age impairment may not have been published. Moreover, many of the results suggesting impairment are based on small sample size and/or have a very small effect size. We have also included these new references from the tDCS papers you suggested, hopefully to make the mixed nature of the findings a bit clearer.**

5. Please provide exact numbers of right- and left-handed participants and make it clearer that all participants used their right-hand regardless of handedness.

**REPLY: This has been updated in the manuscript. We now provide information on handedness in the participants section (Page 6, Lines 116-120).**

6. Why were the last 9 trials of baseline used for analysis – was there any specific rationale for this?

**REPLY: We assume that participants have to get familiar with the task first, which would happen in the first few trials. The last 9 capture each target exactly 3 times and is a better measure of baseline performance. We now provide this rationale in the manuscript (Page 10, lines 209-211).**

7. Figure 3: The authors state “solid lines represent group means”, however there are also dashed lines representing the means of the young and older children’s groups. This needs correcting to avoid confusion.

**REPLY: We have changed the wording to "solid and dashed lines" in the caption for Figure 3.**

8. Figure 3: The authors should report what each aspect of the box plots represents e.g., boxes indicatie the mean, upper and lower quartiles and the range is shown via error bars.

**REPLY: This has been added to the caption for Figure 3B.**

9. A few times in the manuscript the authors write “the usual learning pattern” (Line 286/7) or “the usual generalization pattern” (Line 331), this should be edited to be more specific.

**REPLY: We have clarified this in the manuscript for both instances.**

10. Line 385: “Not only did all groups also relearn to counter the cursor rotation at a similar rate, but this re-learning was significantly faster the second time participants were exposed to the rotation (known as savings) and, again, was independent of age”. Please remove the final part of this sentence as it repeats the info at the beginning (results were not different across age-groups), or re-draft as desired.

**REPLY: This has been edited to be less redundant (Page 19, lines 404-406): “All groups relearned to counter the cursor rotation at a similar rate, and this was significantly faster than the first time participants were exposed to the rotation. That is, all groups showed similar savings.”**

Reviewer #2: In this paper the authors investigate visuomotor learning abilities throughout the lifespan, by comparing rate of adaptation, extent of learning, rate of unlearning, generalization, and savings, in cohorts of young children, older children, young adults, middle-aged adults, and older adults. They find similar results across all ages groups.

This paper looks to resolve inconsistencies in the literature regarding adaptive capacities across ageing. These inconsistencies likely stem from heterogeneity in paradigms that have been used in the past. The authors opt to simultaneously compare several different tasks that probe adaptation across multiple age groups. This is a daunting task, as it is not at all clear whether all these tasks should be similarly susceptible to ageing, as we still have an incomplete understanding of the extent to which their mechanistic bases are common. In fact, it is not clear whether the hypothesis is that all tasks should necessarily show a similar pattern across ageing or not. In this light, I’m not convinced that this strategy represents a fruitful way to “settle” the issue. The fact that there is no effect of ageing certainly adds to the literature, but is inconsistent with many papers that have reported age-related differences, so the impact of the finding is unclear. How does the present results reconcile the previous findings?

Here are some comments to enhance the paper:
1. Abstract: please add the mean age of the different groups

**REPLY: This has been updated.**

2. Introduction: p5 line 84 “Hegele”

**REPLY: This has been updated.**

3. Methods: The number of ppts per group is quite different. Can you confirm that this did not influence the statistical results?

**REPLY: Of course, having more participants per group would have been ideal, but having the same number of participants per group does not affect the robustness of an ANOVA. Blanco et al. (2017) conducted a Monte Carlo simulation and found that different sized groups did not significantly affect the rate of Type I errors. Plus, we also report Bayesian statistics, where normality is not an issue.**

4. There is a relatively low number of datapoints for many measurements (eg. 9 trials for baseline motor control). Under the presence of variability, this may have limited capacity to detect differences and lead to the null effects. The authors should clearly highlight this limitation.

**REPLY: More data points would make the estimates of the variables we’re interested in somewhat more accurate. However, first and foremost we needed an extremely short task that our youngest participants would also finish. Notice that in the motor adaptation field some studies use a single trial as an estimate as well (often for aftereffects). A lot of (our) previous work uses 3-trial block averages and this is sufficient to detect differences between groups as well. Nevertheless, for estimates of baseline bias, and variability, we think using more data is warranted, so we opted for 9-trial estimates.**

5. Discussion: I have a hard time with the interpretation that baseline reaching accuracy is similar across age groups if variability is greater by 50% in children. I get it from the way the dependent variables are calculated here, but taken more broadly, variability would be expected to influence accuracy.

**REPLY: Sorry if this was unclear. Accuracy is independent of variability, the way we measure this here. Within each participant we take accuracy as the average bias (the mean) and we take variability as spread relative to this mean (the standard deviation). We now explain this a bit better in the Methods (Page 11, lines 211-213). We also provide a brief explanation of this again in the Discussion (Page 20, lines 424-425) and cite Contreras-Vidal et al. (2005), who found a similar result as us, and explain it as less fine-tuned motor control.**

6. The authors do not provide much mechanistic explanation to interpret their findings. P21 line 428 provides one such opportunity: how would fun be related to adaptation? If the authors bring this up, do they have literature to back it up? P22 line 451 provides another: how would task quality, and environment, be related to adaptation? Overall this paper would benefit from being less observational/descriptive, and more mechanistically-inspired, hypothesis-driven.

**REPLY: Thanks for the suggestion. We now reference the OPTIMAL theory of motor learning presented by Wulf & Lewthwaite (2016), who argue that increased attention and motivation can strengthen the coupling of goals and actions, leading to enhanced performance (Page 22, lines 458-460). We now expand upon our reasoning on page 23, by suggesting that having novel, gamified stimuli, and positive affect (ie. having fun in a familiar environment) are how we have enhanced attention and motivation, respectively, which are central to this theory.**

7. P25 line 529: It is not clear why the authors would not expect explicit processes in their task, as it has been well documented for such perturbation magnitude (45deg).

**REPLY: You’re right, this is unclear. Another reviewer suggested bringing in another study (Ruitenberg et al., 2023) that uses a 45° rotation and tests a large set of participants across the lifespan. They found slower rates of early adaptation, but not late adaptation, in children and older adults, which they attributed to impaired explicit processes. We have now made several comparisons to this study throughout our discussion section, but still want to make it clear that we are not attempting to distinguish between implicit/explicit contributions here (which is why we have refrained from referring to our adaptation as implicit throughout). We altered the tone here a bit, to acknowledge the *possibility* that we have found no differences in explicit adaptation, but that we cannot speculate further on this issue (Page 24, lines 510-511).**

Reviewer #3: This manuscript describes a study looking at visuomotor learning across the lifespan. It groups participants into young children, older children, young adults, middle-aged adults, and older adults. Participants had to adapt to a visuomotor rotation during a gamified computer/mouse task. The results indicate that age does not impact rate of adaptation, rate of unlearning, generalization, or same-day re-learning. The manuscript is well written and adds important insight into visuomotor learning (especially by including children). With that said, I have several comments that should be addressed.


1. What is the rationale for the specific age ranges to separate age groups? In addition, why are late 20-year-olds part of the middle age group? Typically, middle age is between 40 and 60 (give or take). The choice of age ranges requires justification.

**REPLY: We agree that this could be confusing to any reader. Since our main groups of interest were the two groups of children and older adults, we have updated the group name of the middle adult group to be “Adults”. We will keep the young adult group as is since that label accurately reflects the literature.**

2. The authors quantified mouse use among the children. What about older adults, especially those over 70? Many seniors are not computer savvy.

**REPLY: Unfortunately, as mentioned in our participants section (Page 7, lines 130-131) mouse use data was not captured in the same way for other groups; the 3 adult groups only indicated whether they used a mouse when using a computer. We provided the correlation between mouse familiarity and baseline variability (Page 14, lines 285-286) because the youngest group showed increased baseline variability. Part of this can be explained with lower familiarity with using a mouse, in addition to under-developed fine motor skills. We found that older adults’ variability is comparable to that of most other groups. This suggests that neither fine-motor skills (needed for controlling the mouse), nor mouse familiarity (or computer savviness”) is modulating the older adults’ performance in the main task.**

3. Line 202: why the last 9, and not 10?

**REPLY: Another reviewer raised this question, which is why we now explain this in the manuscript. During the first 6 trials participants were probably still getting familiar with the task. The last 9 capture each target exactly 3 times and are thought to be a better measure of baseline performance. This has been added in on Page 10 and lines 208-210.**

4. For the mixed ANOVAs, how were repeated measures within-participants handled? In JASP, was the repeated measures ANOVA function used? It isn’t clear.

**REPLY: Yes, we used the repeated measures function in JASP and defined things like target or block as within-subjects factors and group as a between subjects factor. We have clarified this in the data analysis section (Page 10, lines 196-198).**

5. Aftereffects: given that visual feedback is available during the washout block, one would expect changes driven by this feedback. The authors do discuss this in a few places in the discussion section. However, for testing aftereffects, why not use the first trial only? This would be the “true” aftereffect. An average of the first 3 trials seems inappropriate for that aspect of the analysis.

**REPLY: We agree that this was not ideal, however, since we wanted to look at generalization, this meant including some additional target locations and they were pseudo-randomly presented to participants during this phase. This meant that most of the participants (2/3) did not reach to the trained target during the first washout trial, but still reached to all three targets in the first three trials. We did fit a decay function to this data and see no qualitative differences between the two approaches. We think that using this computational approach in the main text of the publication would make it less readable to some of our readers who are interested in aging but may not be experts in motor learning; we chose to go with a more straightforward analysis to keep the paper more accessible. However, for readers who find this valuable we now include this analysis in a supplementary file.**

6. Lines 242-247: Can this really be called a rate?

**REPLY: It’s true that this is not the best measure of rate, but this method of calculating rate has been used in other published studies (Seidler, 2007a; 2007b; Cressman et al., 2010). As we explained in our response to your previous comment, we have fitted a function to this data, and included this information in a supplementary file which is referenced in the methods for interested readers.**

7. Some of the Bayes Factors are between 0.33 and 3, and thus anecdotal. However, values close to 1 might suggest the sample size of the groups is too small to detect differences. Have the authors considered this?

**REPLY: An anecdotal result means that the data is not informative about any differences between the null and alternative hypothesis. It is possible that with more participants, we'd still find an anecdotal result (which could be the most likely outcome given our already fairly large sample size), or we could find support for one of the hypotheses; we might find a significant, but probably small difference with limited real-life meaning, or support for the null hypothesis, suggesting there is indeed no differences between the age groups. It would be hard to say a priori, which of these three would happen.**

8. Given the large number of participants overall, the authors should consider running correlations between age and different learning performance measures. Age group might not have an effect, but these correlations might suggest age does a play a role (if significant). I believe this would be a worthwhile analysis, regardless of the outcome.

**REPLY: We were considering if this would be worthwhile and did run a few out of curiosity. We found a significant correlation between age and accuracy (r = 0.176, p = 0.044) which might suggest that as people get older, they become more accurate. We found a significant correlation between age and variability (r = -0.209, p = 0.017) which might suggest that as people get older, they become less variable. We found a significant correlation between age and learning extent in the last block of training (r = 0.178, p = 0.042) which suggests that as people get older, they learn to counter the rotation a bit more. We did not find a significant correlation between age and our measure for aftereffects (first block of washout) (r = -0.034, p = 0.697), suggesting age is not related to implicit learning. However, for the effects which are significant, the effect sizes are small, and we question if reporting these correlations would enhance the manuscript. Additionally, we suspect the fits may not be linear, as we have shown with the additional scatter plots we included for baseline accuracy/precision in Figure 2, which is causing further hesitation to venture forward with this idea.**
